# Safe Listening at Venues and Events with Amplified Music — United States, 2022

**DOI:** 10.15585/mmwr.mm7213a3

**Published:** 2023-03-31

**Authors:** John Eichwald, Christa L. Themann, Franco Scinicariello

**Affiliations:** ^1^Office of Science, National Center for Environmental Health, CDC; ^2^Noise and Bioacoustics Team, Division of Field Studies and Engineering, National Institute for Occupational Safety and Health, CDC; ^3^Office of Innovation and Analytics, Agency for Toxic Substances and Disease Registry, CDC.

Nearly one in four (24.4%) U.S. adults aged 20–69 years show evidence of noise-induced hearing loss (*1*). Among those reporting exposure to noise outside of work, 19.9% showed possible noise-induced hearing loss. Exposure to non–job-related noise can be substantial (*2*). Loud music from personal listening devices and entertainment venues might place more than 1 billion teenagers and young adults at risk for hearing loss worldwide (*3*). Early noise exposure might increase the risk for age-related hearing loss later in life (*4*). CDC analyzed data from the 2022 FallStyles survey (conducted by Porter Novelli via the Ipsos’ KnowledgePanel) on U.S. adult perceptions regarding preventing hearing loss from amplified music at venues or events. More than one half of U.S. adults agreed with one or more of the following protective actions: limiting sound levels, posting warning signs, and using hearing protection when music at such events reaches potentially hazardous levels. Hearing and other health professionals can make use of existing materials available from the World Health Organization (WHO), CDC, and other professional organizations to raise awareness about noise risks and promote protective behaviors.

The 2022 FallStyles survey, conducted during September 1–24, is a nationally representative internet panel comprising 4,514 noninstitutionalized adults aged ≥18 years. The response rate was 78.1%. Results were weighted to the March 2021 supplement of the U.S. Current Population Survey proportions on eight selected demographic variables: sex, age, household income, race and ethnicity, household size, highest level of educational attainment, U.S. Census Bureau region, and metropolitan residency status (living in or near an urbanized area with a population of ≥50,000). Panel members were recruited by mail, using probability-based sampling by address to reach respondents regardless of whether they had landline telephones or Internet access.* If needed, households were provided a laptop or tablet computer and Internet access. Personal identifiers were not included in the data file. Panelists who completed the survey received cash-equivalent rewards worth approximately $5.

Respondents were asked three questions about sound levels at both indoor and outdoor recreational venues and events at which enjoyment of amplified music was a central purpose of attendance. Respondents were asked how much they agreed or disagreed with each of the following statements: “Sound levels at venues or events should be limited to reduce risk of hearing loss”; “Warning signs should be posted if sound at a venue or event could exceed safe levels”; and “I would wear hearing protection if it was provided when sound at a venue or event could exceed safe levels.” Participants indicated their responses on a 5-point Likert scale (strongly agree, agree, neither agree nor disagree, disagree, or strongly disagree). Answers were combined into three categories: 1) strongly agree or agree (agree), 2) neither agree nor disagree, and 3) disagree or strongly disagree (disagree). Multinomial logistic regression was used to calculate adjusted odds ratios (aORs), 95% CIs, and p-values. The following covariates were all included in the model: sex (male or female), age, race and ethnicity, educational attainment, household income, U.S. Census Bureau region of residence, and metropolitan residency status. This analysis was conducted using SAS statistical software (version 9.4; SAS Institute); p-values <0.05 were considered statistically significant. This activity was reviewed by CDC and was conducted consistent with applicable federal law and CDC policy.^†^

Most respondents were female (50.9%), and 62.8% were non-Hispanic White (White) (Table 1). More than one half of respondents (54.1%) agreed that sound levels should be limited at venues or events to reduce risk of hearing loss (Figure); 75.4% agreed that warning signs should be posted if sound at a venue or event could exceed safe levels, and 61.2% agreed that they would wear hearing protection if it was provided when sound at a venue or event could exceed safe levels.

**TABLE 1:** Selected characteristics of surveyed adults aged ≥18 years — Porter Novelli FallStyles survey, United States, 2022

Characteristic	Unweighted no.	Weighted no.	Total respondents weighted, % (95% CI)
**Sex**			
Male	1,788	1,730	**49.1 (47.1–51.0)**
Female	1,738	1,796	**50.9 (49.0–52.9)**
**Age group, yrs, quartiles **			
18–32	450	896	**25.4 (23.4–27.4)**
33–47	685	851	**24.1 (22.4–25.8)**
48–62	650	873	**24.8 (23.2–26.3)**
≥63	1,441	906	**25.7 (24.3–27.1)**
Race and ethnicity*			
Black or African American, non-Hispanic	314	421	**11.9 (10.6–13.3)**
White, non-Hispanic	2,576	2,213	**62.8 (60.8–64.8)**
Hispanic or Latino	375	589	**16.7 (15.0–18.4)**
Other or multiple races, non-Hispanic	261	303	**8.6 (7.4–9.8)**
**Education**			
No high school diploma	181	330	**9.4 (7.9–10.8)**
High school diploma	873	999	**28.3 (26.5–30.1)**
Some college or associate degree	991	957	**27.1 (25.5–28.8)**
Bachelor's degree or higher	1,481	1,240	**35.2 (33.4–36.9)**
**Household income, USD**			
<25,000	383	450	**12.8 (11.4–14.2)**
25,000–74,999	1,098	1,175	**33.3 (31.5–35.2)**
75,000–149,999	1,210	1,097	**31.1 (29.4–32.8)**
≥150,000	835	804	**22.8 (21.2–24.4)**
**U.S. Census Bureau region of residence^†^**			
Northeast	668	608	**17.3 (15.9–18.7)**
Midwest	791	727	**20.6 (19.1–22.6)**
South	1,246	1,347	**38.2 (36.3–40.1)**
West	821	844	**23.9 (22.2–25.6)**
**Metropolitan residency status^§^**			
Nonmetropolitan	438	469	**13.3 (12.0–14.6)**
Metropolitan	3,088	3,057	**86.7 (85.4–88.0)**

**Abbreviation:** USD = U.S. dollars.

* Persons who identified as Black or African American, White, or other or multiple races were all non-Hispanic. Persons who identified as Hispanic might be of any race.

^†^ https://www2.census.gov/geo/pdfs/maps-data/maps/reference/us_regdiv.pdf

^§^ Metropolitan residency status is defined as living in or near an urbanized area with a population of ≥50,000.

**FIGURE:**
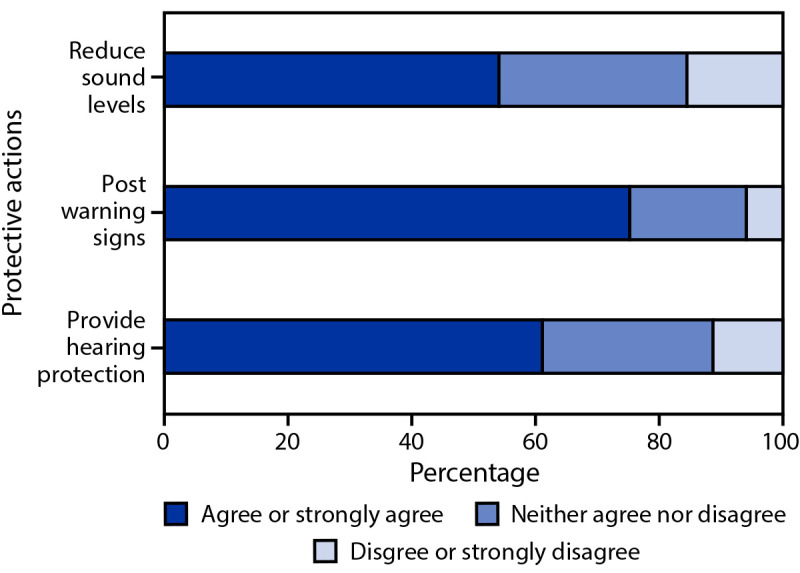
Percentage of agreement or disagreement among adults aged ≥18 years about actions* to protect hearing at indoor or outdoor recreational venues and events at which enjoyment of amplified music is a central purpose of attendance — Porter Novelli FallStyles survey, United States, 2022 * Respondents were asked how much they agreed or disagreed with the following statements: 1) “Sound levels at venues or events should be limited to reduce risk of hearing loss”; 2) “Warning signs should be posted if sound at a venue or event could exceed safe levels”; and 3) “I would wear hearing protection if it was provided when sound at a venue or event could exceed safe levels.”

After adjusting for multiple covariates, women agreed significantly more often than did men that sound levels should be limited and that warning signs should be posted (aOR = 1.2 and 1.5, respectively) (Table 2). Respondents aged ≥63 years agreed significantly more often than did younger adults that sound levels should be limited (2.3), warning signs should be posted (1.4), and that they would wear hearing protection if provided (1.4). However, adults aged 33–47 years and 48–62 years agreed significantly less often that warning signs should be posted (0.6 and 0.7, respectively). Compared with White adults, non-Hispanic Black or African American (Black) adults agreed significantly less often (0.8) with limiting sound levels. Hispanic or Latino (Hispanic) adults agreed significantly more often with displaying warning signs (1.3).

**TABLE 2:** Adjusted odds ratios comparing characteristics of adults who agree with event and venue noise level reduction actions with those of adults who neither agree nor disagree — Porter Novelli FallStyles survey, United States, 2022

Characteristic	Agreement,* aOR^†^ (95% CI)		
	Sound levels at venues or events should be limited to reduce risk of hearing loss^§^	Warning signs should be posted if sound at a venue or event could exceed safe levels^¶^	I would wear hearing protection if it was provided when sound at a venue or event could exceed safe levels**
**Sex**			
Male	Ref	Ref	Ref
Female	1.2 (1.0–1.4)^††^	1.5 (1.3–1.8)^††^	1.1 (0.9–1.3)
**Age group, yrs, quartiles**			
18–32	Ref	Ref	Ref
33–47	1.0 (0.9–1.3)	0.6 (0.5–0.8)^††^	0.8 (0.6–1.0)
48–62	1.2 (0.9–1.5)	0.7 (0.5–0.9)^††^	1.0 (0.8–1.2)
≥63	2.3 (1.8–2.8)^††^	1.4 (1.1–1.8)^††^	1.4 (1.1–1.7)^††^
**Race and ethnicity** ^§§^			
Black or African American, non-Hispanic	0.8 (0.6–1.0)^††^	0.8 (0.6–1.0)	1.0 (0.8–1.3)
White, non-Hispanic	Ref	Ref	Ref
Hispanic or Latino	1.0 (0.8–1.3)	1.3 (1.0–1.7)^††^	1.1 (0.9–1.3)
Other or multiple races, non-Hispanic	1.0 (0.8–1.3)	0.8 (0.6–1.2)	0.9 (0.7–1.2)
**Education**			
No high school diploma	Ref	Ref	Ref
High school diploma	1.5 (1.1–2.0)^††^	1.1 (0.8–1.5)	1.5 (1.1–1.9)^††^
Some college or associate degree	2.1 (1.5–2.8)^††^	1.5 (1.1–2.1)^††^	1.9 (1.4–2.6)^††^
Bachelor's degree or higher	3.1 (2.3–4.2)^††^	2.4 (1.7–3.4)^††^	2.8 (2.1–3.8)^††^
**Household income, USD**			
<25,000	Ref	Ref	Ref
25,000–74,999	1.1 (0.9–1.4)	1.2 (0.9–1.6)	1.0 (0.8–1.3)
75,000–149,999	1.0 (0.7–1.3)	1.1 (0.8–1.4)	0.9 (0.7–1.1)
≥150,000	1.1 (0.8–1.4)	1.4 (1.0–2.0)^††^	1.2 (0.9–1.7)
**U.S. Census Bureau region of residence** ^¶¶^			
Northeast	Ref	Ref	Ref
Midwest	0.9 (0.7–1.1)	1.0 (0.8–1.4)	1.0 (0.8–1.3)
South	0.9 (0.7–1.1)	0.9 (0.7–1.2)	0.9 (0.7–1.2)
West	0.9 (0.7–1.1)	0.9 (0.7–1.2)	1.0 (0.7–1.2)
Metropolitan residency status***			
Nonmetropolitan	Ref	Ref	Ref
Metropolitan	1.1 (0.8–1.3)	1.5 (1.1–2.0)^††^	1.0 (0.8–1.3)

**Abbreviations**: aOR = adjusted odds ratio; Ref = referent group; USD = U.S. dollars.

* “Agreement” includes both “strongly agree” and “agree” responses.

^†^ aORs and 95% CIs calculated using multinomial logistic regression.

^§^ Panelists were asked to indicate level of agreement with the following statement: “Sound levels at venues or events should be limited to reduce risk of hearing loss.”

^¶^ Panelists were asked to indicate level of agreement with the following statement: “Warning signs should be posted if sound at a venue or event could exceed safe levels.”

** Panelists were asked to indicate level of agreement with the following statement: “I would wear hearing protection if it was provided when sound at a venue or event could exceed safe levels.”

^††^ Statistical difference at p<0.05 compared with Ref.

^§§^ Persons who identified as Black or African American, White, or other or multiple races were all non-Hispanic. Persons who identified as Hispanic might be of any race.

^¶¶^ https://www2.census.gov/geo/pdfs/maps-data/maps/reference/us_regdiv.pdf

*** Metropolitan residency status is defined as living in or near an urbanized area with a population of ≥50,000.

Agreement with both limiting sound levels and wearing hearing protection progressively increased with the respondent’s level of educational attainment. Compared with those with less than a high school education, adults with a high school diploma, with some college or associate degree, and with a bachelor’s degree or higher agreed significantly more often with limiting sound levels (aOR = 1.5, 2.1, and 3.1, respectively) and wearing hearing protection (1.5, 1.9, and 2.8, respectively). Only adults with some college and those with at least a bachelor’s degree agreed significantly more often than did those with less education that warning signs should be posted in venues and events where sound could exceed safe levels (1.5 and 2.4, respectively).

Compared with respondents who reported an annual household income of <$25,000, those with an income of >$150,000 agreed significantly more often (aOR = 1.4) that warning signs should be posted. U.S. Census Bureau region and metropolitan residency status made little difference in perceptions regarding safe listening to amplified music at venues and events, with the exception of adults in metropolitan areas who agreed significantly that warning signs should be posted (1.5).

## Discussion

The results from this survey indicate that U.S. adults are largely aware of the hazard posed by high sound levels at concerts and other events. More importantly, results indicate an encouraging openness to protective actions, such as limiting sound levels, posting warning signs, and use of hearing protection. More than one half of the respondents agreed that sound levels at venues or events should be limited to reduce risk for hearing loss, approximately three quarters of respondents agreed that warning signs should be posted if sound at a venue or event could exceed safe levels, and approximately three in five respondents agreed they would wear hearing protection if it was provided when sound at a venue or event could exceed safe levels. Survey results suggest targeting educational efforts for the use of hearing protection toward respondents aged <63 years. Raising the awareness among certain demographic groups (e.g., younger persons, Black persons, and Hispanic persons) about limiting sound levels and displaying of warning signs might be warranted.

However, stated intent to take protective action does not always result in the action being taken. In an earlier Porter Novelli Styles survey, approximately 80% of U.S. adults aged ≥18 years reported never or seldom using hearing protection at loud athletic or entertainment events. An additional 10% reported using hearing protection only some or about one half the time (*5*). Interventions focusing on translating intent into behavior are needed. Healthy People 2030, the nation’s public health agenda, includes an objective to increase the proportion of adults who use hearing protection devices when exposed to loud sounds (*6*).

In 2022, WHO published a Global Standard recommending sound levels at venues and events be limited to no more than 100 dB(A) equivalent continuous sound level^§^ over any 15-minute period. The limit was set to reduce “unnecessarily hazardous sound levels” while still allowing for artistic expression and enjoyment of amplified music. WHO acknowledged the limit “does not, and cannot, eliminate all risk of an individual audience member suffering sound-induced hearing injury,” particularly among those who frequently attend loud music events (*7*). WHO’s standard provides examples of preventive actions to reduce risk for hearing loss for audience members. These include those surveyed in this report: limiting sound levels, posting warning signs when sound could exceed safe levels, and providing hearing protection, such as earplugs, with appropriate instructions for audience members.

The findings in this report are subject to at least four limitations. First, the data obtained in this survey were self-reported. Second, the survey relied on respondents’ perceptions of loudness and the risk for hearing loss. Third, respondents’ perceptions might be influenced by their experience of attending such events, but data on these experiences (e.g., whether or how often respondents attended events with amplified music) were not collected. Finally, although survey responses were weighted by the U.S. demographic characteristics, how accurately this weighting has corrected any bias in this internet panel sample remains unknown.

Music-induced hearing loss is entirely preventable. Hearing and other health professionals can make use of existing materials available from WHO (https://www.who.int/activities/making-listening-safe), CDC (https://www.cdc.gov/nceh/hearing_loss/toolkit), and a variety of professional organizations (e.g., http://dangerousdecibels.org/ and https://hearinghealthfoundation.org/keeplistening) to raise awareness of noise-related risks and promote protective behaviors, such as lowering the volume, using hearing protection, and taking breaks from noisy activities. Interventions should focus on helping persons understand the risks of high sound levels and managing their exposures so they can enjoy music for a lifetime without the debilitating effects of hearing loss.

**Summary**

**What is already known about this topic?**

Exposure to loud music from personal listening devices and entertainment venues can pose a risk to hearing; nearly 25% of U.S. adults aged 20–69 years show evidence of noise-induced hearing loss.

**What is added by this report?**

More than one half of U.S. adults aged ≥18 years are open to actions being taken at events and venues with amplified music to protect their hearing, such as limiting sound levels, posting of warning signs, and using hearing protection when provided.

**What are the implications for public health practice?**

Health care practitioners can help persons understand their risks from high sound levels and manage their exposures. Resources are available to help raise awareness of noise risks and promote protective behaviors.
